# Assessment of Heterotrophic Growth Supported by Soluble Microbial Products in Anammox Biofilm using Multidimensional Modeling

**DOI:** 10.1038/srep27576

**Published:** 2016-06-07

**Authors:** Yiwen Liu, Jing Sun, Lai Peng, Dongbo Wang, Xiaohu Dai, Bing-Jie Ni

**Affiliations:** 1 State Key Laboratory of Pollution Control and Resources Reuse, College of Environmental Science and Engineering, Tongji University, Shanghai 200092, P. R. China; 2 Centre for Technology in Water and Wastewater, School of Civil and Environmental Engineering, University of Technology Sydney, Sydney, NSW 2007, Australia; 3 Laboratory of Microbial Ecology and Technology (LabMET), Ghent University, Coupure Links 653, 9000 Ghent, Belgium; 4 College of Environmental Science and Engineering, Hunan University, Changsha 410082, China; 5 Key Laboratory of Environmental Biology and Pollution Control (Hunan University), Ministry of Education, Changsha 410082, China

## Abstract

Anaerobic ammonium oxidation (anammox) is known to autotrophically convert ammonium to dinitrogen gas with nitrite as the electron acceptor, but little is known about their released microbial products and how these are relative to heterotrophic growth in anammox system. In this work, we applied a mathematical model to assess the heterotrophic growth supported by three key microbial products produced by bacteria in anammox biofilm (utilization associated products (UAP), biomass associated products (BAP), and decay released substrate). Both One-dimensional and two-dimensional numerical biofilm models were developed to describe the development of anammox biofilm as a function of the multiple bacteria–substrate interactions. Model simulations show that UAP of anammox is the main organic carbon source for heterotrophs. Heterotrophs are mainly dominant at the surface of the anammox biofilm with small fraction inside the biofilm. 1-D model is sufficient to describe the main substrate concentrations/fluxes within the anammox biofilm, while the 2-D model can give a more detailed biomass distribution. The heterotrophic growth on UAP is mainly present at the outside of anammox biofilm, their growth on BAP (HetB) are present throughout the biofilm, while the growth on decay released substrate (HetD) is mainly located in the inner layers of the biofilm.

The anaerobic ammonia oxidation (anammox) process is a promising autotrophic nitrogen removal technology, during which ammonia is oxidized to nitrogen gas with nitrite as the electron acceptor^1^,^2^,^3^,^4^,^5^,^6^,^7^. This autotrophic process has a lower oxygen demand and a lower sludge production and does not require external carbon source^8^,^9^,^10^, contributing to substantially lower operation costs compared to the conventional denitrification systems^11^. The anammox bacteria present a slow growth rate with a doubling time of ca. 7–11 days^12^,^13^,^14^. The yield of the anammox bacteria has been reported to be 0.066 C-mol biomass per mol ammonium consumed, and the maximum ammonium consumption rate is ca. 45 nmol mg^−1^ protein min^−1^ 12. Thus, efficient biomass retention is indispensable for retaining the anammox bacteria within the reactor throughout the cultivation. An effective way of immobilizing anammox bacteria in wastewater treatment processes is that the bacteria are forced to grow as biofilms, which consists of an aggregation of cells and abiotic particulates within an organic polymeric matrix of microbial origin such as extracellular polymeric substances (EPS) and soluble microbial products (SMP) produced during normal metabolic activity^15^,^16^,^17^,^18^. SMP capture the natural products of bacterial growth (utilization-associated products, UAP) and hydrolysis (biomass-associated products, BAP), allowing for an interaction among bacterial species^17^,^18^,^19^. Usually, the UAP exhibited characteristics of carbonaceous compounds with a molecular weight lower than 1 kDa, while the BAP consisted mainly of macromolecules with a molecular weight higher than 1 kDa^20^. Therefore, their impacts on biological wastewater systems differ substantially and are considered separately^17^.

Previous studies have confirmed ecophysiological interaction between autotrophic and heterotrophic bacteria in autotrophic suspended cultures^21^ and biofilms^22^,^23^ grown without external organic carbon substrates. Coexistence of a high level of heterotrophic bacteria with anammox bacteria has also been reported in anammox biofilm^18^. The microbial products (such as SMP) have been shown to be the energy and carbon sources for the heterotrophic growth, although no other organic carbon substrates are present in feeding solutions^24^. However, there are a limited number of studies documenting about organic substrate uptake patterns of heterotrophic bacteria detected in autotrophic biofilms. Kindaichi *et al.*^22^ reported that an autotrophic nitrifying biofilm was consisted of 50% nitrifying bacteria and 50% heterotrophic bacteria, and the distribution was as follows: members of the α-*Proteobacteria*, 23%; γ-*Proteobacteria*, 13%; green nonsulfur bacteria, 9%; *Cytophaga-Flavobacterium-Bacteroides* (CFB) division, 2%. O’Sullivan *et al.*^25^ found that some members of CFB utilize various biomacromolecules, such as cellulose, proteins, DNA, chitin and lipids, which may be accumulated in biofilms. Cottrell and Kirchman^26^ demonstrated that the a- and g-*Proteobacteria* in marine environments uptake amino acids. Furthermore, Okabe *et al.*^23^ revealed that the members of the *Cytophaga*-*Flavobacterium* cluster gradually utilized ^14^C-labeled products in the culture with ammonium addition where nitrifying bacteria grew. This result revealed that these bacteria preferentially utilized UAP of nitrifying bacteria. The member of the *Chloroflexi* utilizes the cell materials of decaying nitrifying bacteria (i.e., decay released products), and members of the α-*Proteobacteria* and γ-*Proteobacteria* uptake low-molecular-weight organic matter generated by hydrolysis of EPS (i.e., BAP).

Since community structure in the anammox biofilm is determined by a complex interplay of various factors including the concentration of chemical species, presence of other bacteria and their physiology, mathematical modeling provides a logical framework for the exploration of processes within biofilm^19^,^27^,^28^. Therefore, in the present work, 1-D and 2-D numerical biofilm models highlighting ecophysiological interaction between anammox and heterotrophic bacteria within the anammox biofilm are applied to evaluate and characterize the heterotrophic growth supported by microbial products in anammox biofilm.

## Results and Discussion

### 1-D modeling results

**Figure 1:**
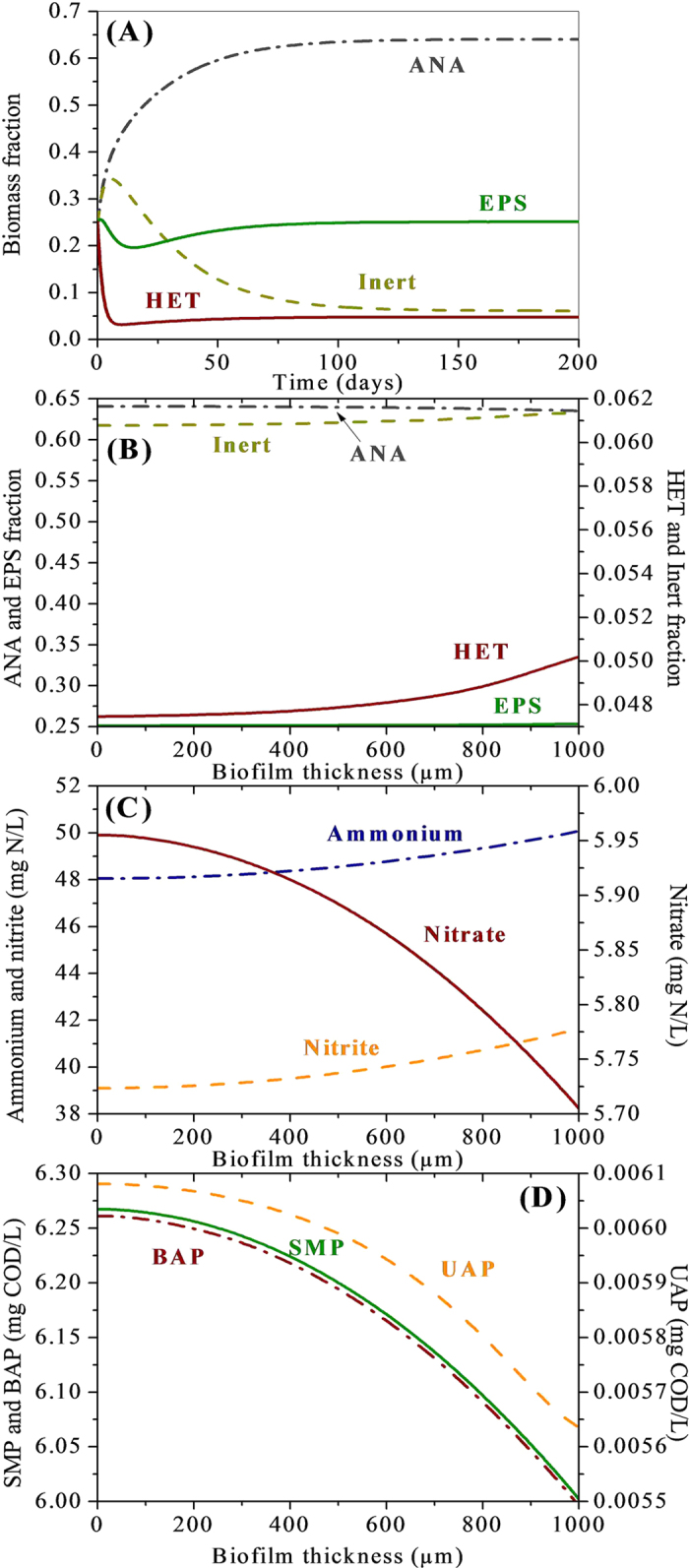
One-dimensional simulation results for the anammox biofilm. Biomass fractions during the 200-day biofilm development (**A**), and population distributions (**B**), substrate profiles (**C**), SMP (including UAP and BAP) profiles (**D**) at steady state in the anammox biofilm. The applied biofilm thickness and influent ammonium and nitrite concentration were 1000 μm, 100 mg N L^−1^, and 100 mg N L^−1^, respectively.

The 1-D simulation is first conducted for 200 days, which allow the system to come to a global steady state for all the soluble and solid components in the anammox biofilm. Figure 1A presents the simulation results for the biomass fractions of X_ANA_, X_HET_, EPS, and X_I_ out to 200 days of biofilm reactor operation. For the active biomass components (Fig. 1A), the active anammox bacteria and heterotrophs take at least 100 days to reach a steady state. The biomass in the anammox biofilm is comprised of anammox bacteria at 60%, heterotrophs at 10%, X_I_ at 10%, and EPS at 20% at global steady state, suggesting that a significant amount of heterotrophic growth are present in this anammox biofilm. Microbial products (UAP, BAP and readily biodegradable substrate (Ss)), not contained in the feed solution, are the energy and carbon sources for the growth of heterotrophic bacteria.

Typical simulation results of depth profiles in the anammox biofilm are shown in Fig. 1B–D. The heterotrophic bacteria are dominant at the surface of the biofilm from 600–1000 μm while anammox bacteria are dominant throughout the whole biofilm structure (Fig. 1B). Although heterotrophic bacteria can use nitrite as the terminal electron acceptor for their growth, heterotrophic bacteria are outcompeted by anammox bacteria at the base of the biofilm (Fig. 1B), likely due to their relative low affinity for nitrite as compared to anammox bacteria and also for SMP when utilizing nitrite^27^. The ammonium and nitrite profiles in the anammox biofilm can be seen in Fig. 1C, which are very close to those in the bulk liquid. The nitrate concentrations at the surface of the biofilm from 600–1000 μm (zone with heterotrophs domination) are lower than those at the base of the biofilm (Fig. 1C) because the produced nitrate by anammox can be utilized as electron acceptor for heterotrophic growth. The SMP profiles within the anammox biofilm are shown in Fig. 1D, together with the UAP and BAP profiles. The total SMP concentrations (both UAP and BAP) in the zone with heterotrophs domination are lower than those at the base of the biofilm. This simulation result again suggested that microbial products should be the substrates for the growth of heterotrophic bacteria. In addition, simulation results show that the BAP account for the major remained SMP in the whole biofilm. The UAP concentrations are much lower than BAP, indicating that the heterotrophs prefer to utilize UAP as carbon source for their growth. This leads to the necessity to investigate the relative importance of UAP and BAP for the heterotrophic growth in anammox biofilm.

### 2-D modeling results

**Figure 2:**
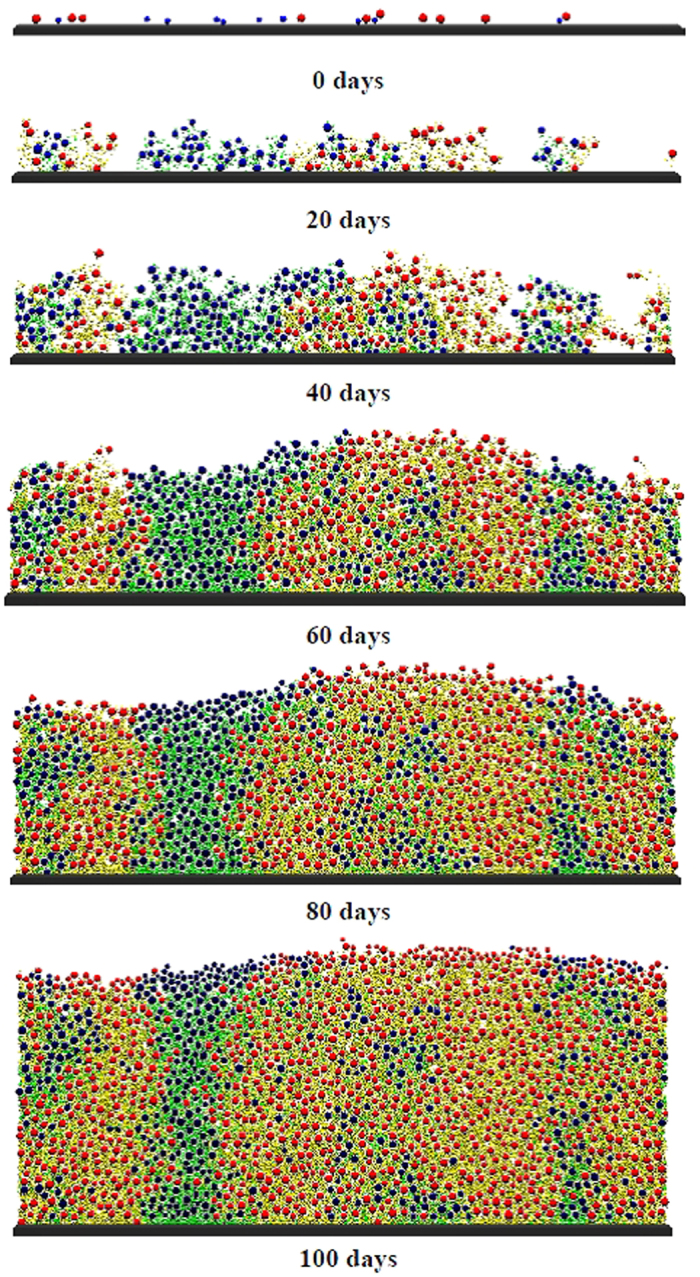
Two-dimensional simulation results of spatial distributions of microbial populations and EPS in an anammox biofilm at different moments in time. In this visualization the biomass particles with a size of about 2 mm each have colors corresponding to their microbial composition (red: anammox bacteria; blue: heterotrophs; yellow: EPS from anammox bacteria; and green: EPS from heterotrophs).

The 1-D models hold the advantages of simplicity and faster computations, while the 2-D models give a more specific biofilm structure. Both types of models used identical biological processes model and parameters (see Tables S1–S3). Figure 2 shows the 2-D model simulation results of microbial distributions within anammox biofilm developing along time. At early stages of the biofilm, due to sufficient transfer of nitrite and ammonia from bulk liquid, the biofilm is mainly constituted of anammox bacteria due to the large particle size (0 days in Fig. 2). As the increase of the biofilm thickness, an anoxic heterotrophic growth zone is established at the inner layer of the biofilm (40 days in Fig. 2). This condition is preferable for heterotrophs to uptake the excreted organic material (i.e., SMP) as electron donors. Therefore, at the later stage of biofilm formation (80 days in Fig. 2), heterotrophs mainly exist at the outer layer of the biofilm with some fractions in the inner part of the biofilm, while anammox bacteria dominate throughout the whole biofilm. At later stage, throughout of the biofilm there becomes dominated by EPS produced by the anammox bacteria (100 days in Fig. 2). These global 2-D simulation results are similar to those of 1-D simulations (Fig. 1). As the 2-D model includes stochastic elements (i.e. the initial spatial distribution of microbial types and the direction of cell division and EPS production), results obtained with identical sets of parameters are not entirely identical, but very similar. However, the 2-D models can present a more detailed biofilm structure with 2-D biomass distributions (Fig. 2).

The concentrations of N-species in the bulk liquid along time in the 2-D simulations are also comparable to 1-D simulation results. For initial biofilm developing time (before day 40), the concentrations calculated with 1-D and 2-D models are identical (data not shown). Although the computed steady state values of N-species concentrations in the bulk liquid are similar between 1-D and 2-D models (from approximately day 100 on), the 2-D model results present some fluctuation around the average value (data not shown). This is because there are spatially developed colonies or cell clusters of one microbial type in 2-D model, different from the microbial distribution in uniform layers in 1-D model (Fig. 2). After the biofilm has reached the maximum imposed thickness, in 2-D model whole colonies can be rapidly detached from the biofilm surface, while in 1-D model such detachment process is completely smooth. For example, if a large piece of anammox bacteria is washed off from the biofilm at a short interval, the ammonium and nitrite concentrations will suddenly increase while the nitrate will decrease. Due to these fluctuations, a true steady state is never achieved in 2-D simulations, neither for solutes nor for microbial compositions. Steady values can only be mentioned by comparing values averaged over an interval of ca. 5–10 days. These results present that a 1-D model is adequate to describe the main solute concentrations and fluxes in the anammox biofilm (ammonium, nitrite, and nitrate). However, for low-concentration solutes (i.e., UAP and BAP), the 2-D model is more dependent on the spatial distribution of different microbial communities, which results in different profiles from those computed with a 1-D model.

### Relative importance of UAP and BAP

**Figure 3:**
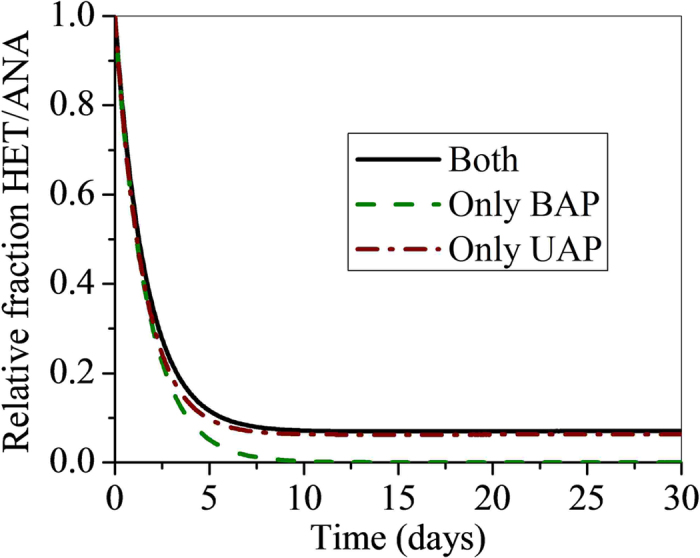
Effects of heterotrophic growth solely on UAP, BAP, or both. Plot of relative fraction of heterotrophs to anammox bacteria as a function of time for growth on UAP alone, BAP alone, and on both SMP types together. For growth only on UAP, the value of μ_H,BAP_ was set to zero, and for growth on BAP alone the value of μ_H,UAP_ was set to zero. It is readily apparent that heterotrophic growth depends almost entirely on utilization of UAP rather than BAP; the only BAP curve yields almost no heterotrophic survival.

Given that both types of SMP have been covered in the model, we evaluated the relative importance of UAP and BAP to heterotrophic growth by first removing heterotrophic growth on first one and then the other SMP component; this was completed by setting either μ_UAP_ or μ_BAP_ to 0, with the other SMP parameters as listed in the in Table S3. The relative importance of UAP and BAP to heterotrophic growth is summarized in Fig. 3, which is the relative fraction of heterotrophic to anammox bacteria (X_HET_/X_ANA_) in the biofilm as a function of time for each case of SMP uptake. It is easily observed from the simulation that heterotrophic growth occurs primarily through metabolism of UAP and very little via BAP metabolism. Such trend is not surprising given the fact that the UAP utilization rate is much larger than the BAP utilization rate (higher than a factor of 18 from Laspidou and Rittmann^16^, Table S3). Also important aspects are the different sources of UAP and BAP within the biofilm: UAP is generated continuously from anammox bacteria growth (at a relatively high specific rate), whereas BAP is produced through hydrolysis of EPS and biomass decay (at a relatively slow specific rate). Thus, we can use this result in summarizing heterotrophic growth on SMP by focusing on UAP parameters.

### Production and utilization kinetics of UAP

The previous section underlines the strong effect UAP may have on the resulting heterotrophs to anammox bacteria fraction; therefore, we explored the influence of the production and utilization kinetic values (UAP formation coefficient f_UAP_ , maximum growth rate of heterotrophs on UAP μ_H,UAP _, and substrate utilization rate of anammox bacteria q_N_) of UAP on the resultant biofilm composition. This was done by setting the biomass affinity constant values for UAP and BAP utilizations (K_UAP_ and K_BAP_ , respectively) very low so that substrate limitation was not a factor. We used K_UAP_ = 0.01 mg COD L^−1^ and K_BAP_ = 0.0085 mg COD L^−1^, 4 orders of magnitude lower than the values used in Laspidou and Rittmann^16^ and small enough that for all but the lowest SMP concentrations the net growth rate did not vary. The ratio of μ_H,UAP_ to μ_H,BAP_ was kept equal to the ratio of these values in Laspidou and Rittmann^16^, with the values used being provided in Table S3.

**Figure 4:**
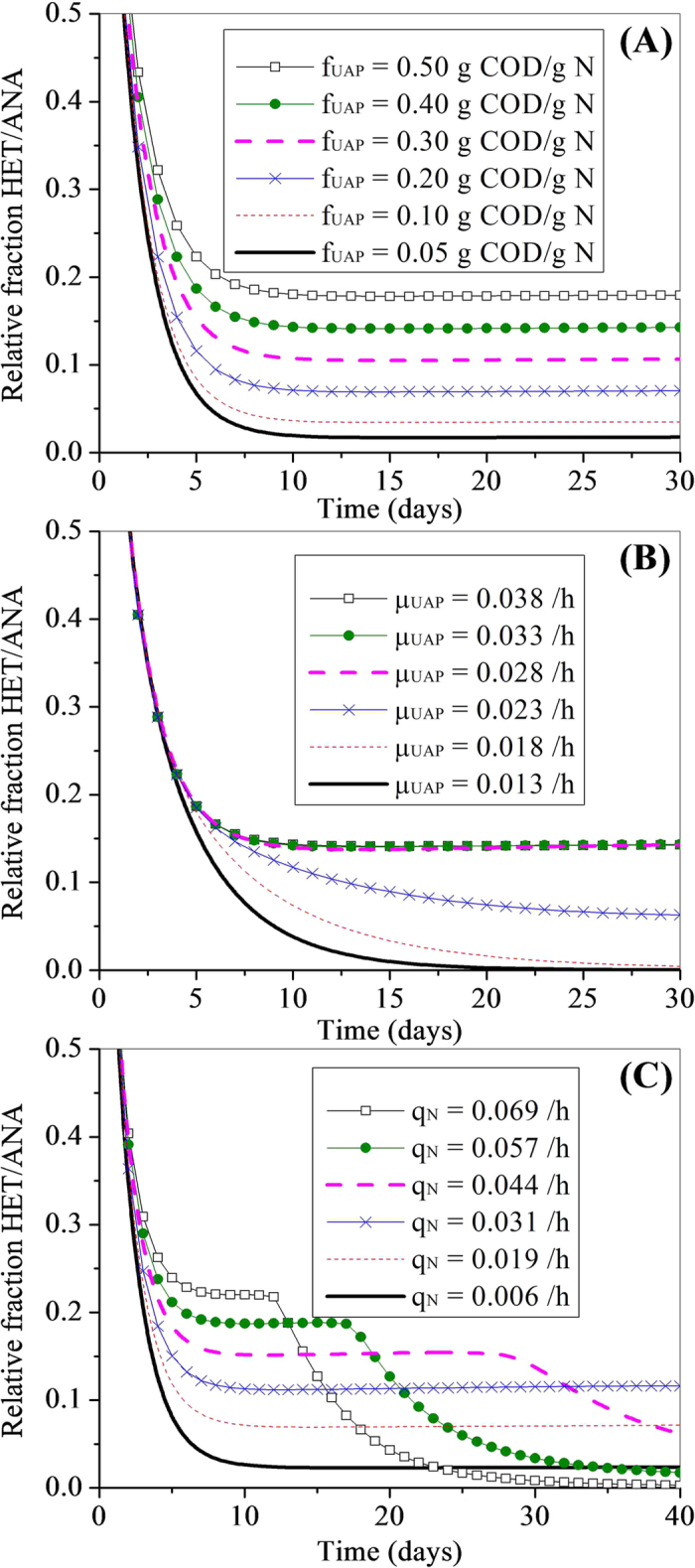
Influence of the production and utilization kinetic values of UAP on the relative fraction of heterotrophs to anammox bacteria as a function of time. (**A**) UAP formation coefficient f_UAP_ , (**B**) maximum growth rate of heterotrophs on UAP μ_H,UAP_ , and (**C**) substrate utilization rate of anammox bacteria q_N_.

Figure 4A presents the relative fraction of heterotrophic bacteria to anammox bacteria (X_HET_/X_ANA_) within the biofilm as a function of time for the different parameter sets of f_UAP_. The relative fraction decreases quickly in the early simulation, but the ratio stabilizes soon after; this decline and stabilization are a fact of the delay between SMP production from the anammox bacteria and its uptake resulting in growth by the heterotrophs. It can be seen that, when substrate limitation is not a factor (i.e., K_UAP_ and K_BAP_ are very low), any of the f_UAP_ values is adequate to yield heterotrophic subsistence on SMP; further, all f_UAP_ values eventually result in the relative fraction of heterotrophs within the biofilm greater than 0.02. In addition, an increase in f_UAP_ value results in an increase in the relative fraction (Fig. 4A). Such an effect is attributed to the fact that f_UAP_ is the yield coefficient for UAP, which is a multiplicator of the total UAP production. Higher UAP production then results in higher heterotrophic growth. Also, it should be noted that after ca. 20 days the ratio between heterotrophs and autotrophs is almost constant, indicating that the species balance has achieved a steady state. This is because of the balance of SMP production and consumption potentials of the anammox and heterotrophic bacteria, respectively.

Figure 4B reveals the relative fraction of heterotrophs to autotrophs as a function of time for these varied-*μ*_*H,UAP*_ simulation. The effect of *μ*_*H,UAP*_ on the relative fraction of heterotrophs to anammox bacteria output is minimal at high values of *μ*_*H,UAP*_ (Fig. 4B). However, as *μ*_*H,UAP*_ is reduced, the relative fraction changes significantly. During the biofilm developing period, at a *μ*_*H,UAP*_ of 0.013 h^−1^, the relative fraction of heterotrophs to anammox bacteria is much lower than the corresponding values at high *μ*_*H,UAP*_ values. This figure shows the same initial decrease in ratio at the beginning, but then the stabilization for only some of the parameter sets. This reveals that, for these utilization rates, only the relative high *μ*_*H,UAP*_ values allow the heterotrophs to subsist in the anammox biofilm.

Figure 4C presents the relative fraction of heterotrophs to anammox bacteria (X_HET_/X_ANA_) in the biofilm as a function of time for the different parameter sets of q_N_. Generally, an increase in q_N_ results in an increase in the relative fraction when q_N_ is lower than 0.031 h^−1^ (Fig. 4C). Such an effect is attributed to the fact that UAP formation rate is proportional to the total substrate consumption rate of anammox. However, further increasing q_N_ has an influence on the model output in an opposite way (Fig. 4C). A higher q_N_ value results in a lower relative fraction of heterotrophs to anammox bacteria. This was associated with the lack of substrates (ammonium and nitrite) in the anaerobic layer limits the UAP production.

### Uptake patterns for organic substrates derived from anammox bacteria

**Figure 5:**
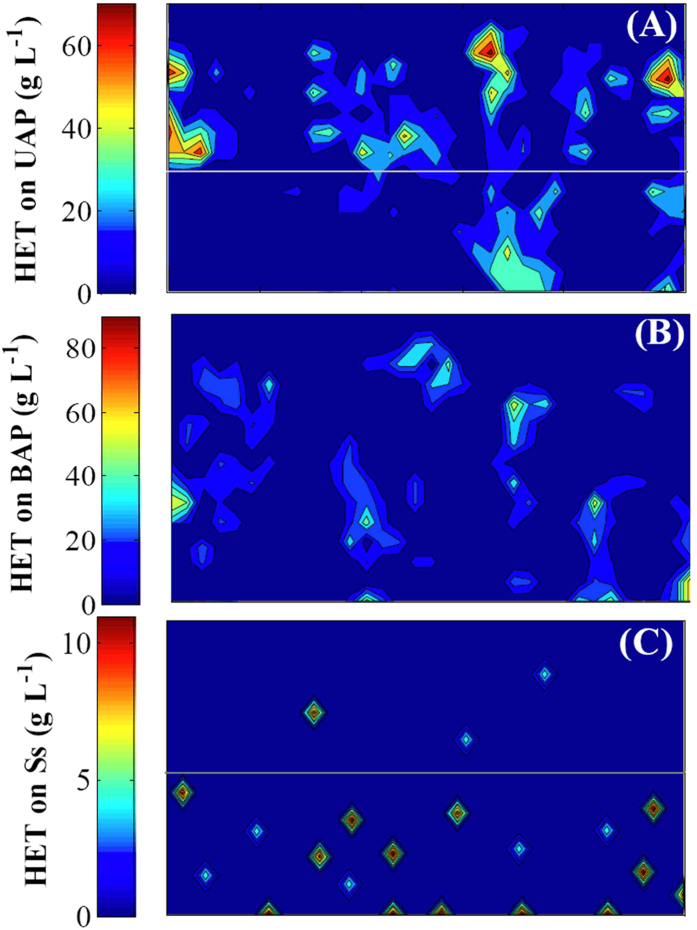
Detailed insight into the spatial localization of HetU (A), HetB (B) and HetD (C) in the anammox biofilm provided by the 2-D simulation at day 100. X-axis is the horizontal length of biofilm, while Y-axis is the vertical depth of biofilm (bottom indicates substratum of the biofilm).

In our modeling approach, organic carbon for the growth of the heterotrophic bacteria is derived from three reactions: anammox growth (UAP), biomass decay (decay released products) and hydrolysis of EPS (BAP). Thus, we integrated three types of heterotrophic bacteria: as HetU, HetB and HetD that utilize UAP, BAP and decay released products respectively. Although those hypotheses are not reflecting all of the bacterial uptake patterns for organic substrates derived from anammox bacteria, the results of this study can be plausibly explained on the basis of hypotheses. Detailed 2-D simulated distribution of heterotrophs in the anammox biofilm is presented in Fig. 5. HetU is located mainly at the outside of anammox biofilm where sufficient UAP is produced by active anammox bacteria (Fig. 5A). HetB are present throughout the biofilm as EPS produced by anammox bacteria spread broadly in biofilm (Fig. 5B). HetD is mainly present in the inner parts of biofilm where decay released products is produced from inactivated anammox bacteria because of depletion of external substrate (Fig. 5C). In addition, the HetU account for more than 65% of the total heterotrophic growth in anammox biofilm while HetD only account for about 5% of the total heterotrophic growth attributed to too low decay rate of anammox bacteria. The simulation results demonstrated that distributions of HetU, HetB and HetD are consistent with those of CFB, *Proteobacteria*, and *Chloroflexi* reported in literature^22^,^23^,^27^. The model simulation results indicate that the properties of anammox biofilm systems resulted from microbial interactions are generally not tractable by current experimental approaches.

### Implications of this work

Mathematical modeling has been proven to be a powerful tool for comprehensively understanding complex microbial processes in biological wastewater treatment^19^,^29^,^30^,^31^,^32^,^33^,^34^,^35^,^36^. In this work, 1-D and 2-D mathematical modeling were applied to assess the heterotrophic bacterial growth on three types of microbial products (UAP, BAP, and decay released substrate) from anammox bacteria in anammox biofilm, during the biotransformation of nitrogen compounds. The model process kinetics have been evaluated and validated by experimental data in previous studies. With this model, we gained significant insights into heterotrophic growth processes on SMP in anammox biofilm systems, which was not available previously. These results provided useful information for the operation of the promising anammox biofilm process for nitrogen removal.

#### Selection of 1-D or 2-D model

The 2-D biofilm modeling approach can be used to more accurately explain microbial interactions such as syntrophy/competition for substrates and space, which are more difficult to be described by 1-D models. However, 1-D models appear to be sufficient to predict solute concentrations and fluxes, and preferable in this case due to their simplicity and computational efficiency.

#### Roles of SMP on heterotrophic growth in anammox biofilms

Considerable amount of heterotrophic growth was revealed to take place in anammox biofilm and the UAP of anammox bacteria was the main organic carbon source for heterotrophs. Heterotrophs were dependent heavily on UAP production and utilization kinetics to sustain themselves as UAP was their major substrate. HetU is present mainly at the outside of anammox biofilm, HetB are present throughout the biofilm, while HetD is mainly present in the inner parts of biofilm. In addition, the HetU account for more than 65% of the total heterotrophic growth in anammox biofilm while HetD only account for about 5% of the total heterotrophic growth. According to these simulation results, future experiments can be appropriately designed and in turn validate current modeling results.

Ideally, the above goal in this study would be achieved if the model could be calibrated using comprehensive datasets. This is unfortunately not possible at present due to the lack of data. We have therefore chosen to employ a well-established model previously developed^18^, with the model process kinetics being evaluated and validated by extensive experimental data. We recognize that without being validated with data, the model predictions are preliminary and remain to be verified. However, we believe the preliminary results will already support our understanding in this area. For example, heterotrophic growth by SMP should be incorporated into modeling anammox biofilm systems in the future. This will be more accurate to describe syntrophic metabolizing between two groups of bacteria for better prediction of process dynamics and effluent quality of the anammox process.

## Materials and Methods

### Biological processes model

**Figure 6:**
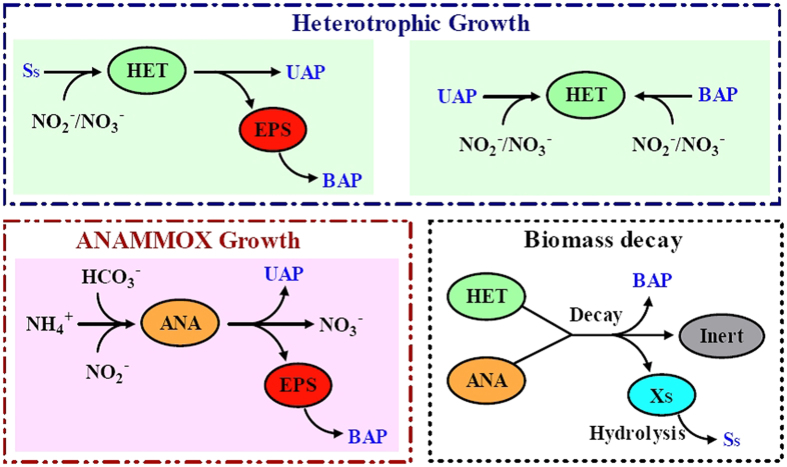
Schematic representation of the bio-reactions carried out in individuals of the two microbial groups and the interactions among the involved components with exchanges of microbial products between anammox bacteria (ANA) and heterotrophs (HET) in anammox system.

The biological processes model developed and validated by Ni *et al.*^18^ was applied, in which the anammox kinetics were extended through integrating with new kinetics of microbial products formation from Anammox process^24^. The model process kinetics, especially the proposed distinct utilization by heterotrophs has been well accepted and widely applied in literature^15^,^16^,^18^,^19^,^21^,^22^,^27^,^28^,^37^. An experimental evaluation on heterotrophic growth on SMP in autotrophic anammox biofilms has been conducted in our previous work^18^, with the generated data including SMP measurement and microbial analysis data, being used for verification of the model applied. The kinetic model for the biological processes considered that organic carbon for the growth of the heterotrophic bacteria was derived from three reactions: anammox bacterial growth (UAP), biomass decay (bacterial decay released substrate (Ss) and BAP)^38^ and hydrolysis of EPS (BAP)^15^. Figure 6 represents the heterotrophic growth on the three types of microbial products (UAP, BAP and Ss) from anammox. It should be pointed that hydrolysis of EPS was assumed to be the only source of BAP according to the unified theory of Laspidou and Rittmann^15^. However, there may be some disagreements in the literature about the BAP formation^32^,^37^,^39^. In addition to the EPS hydrolysis, there are other BAP that might be released into the bulk solution as a result of lysis (i.e., endogenous organics and cell debris) and cell maintenance (i.e., turnover of intracellular components)^40^. In this model, both soluble EPS and biomass decay products were considered to be the sources of BAP.

**Figure 7:**
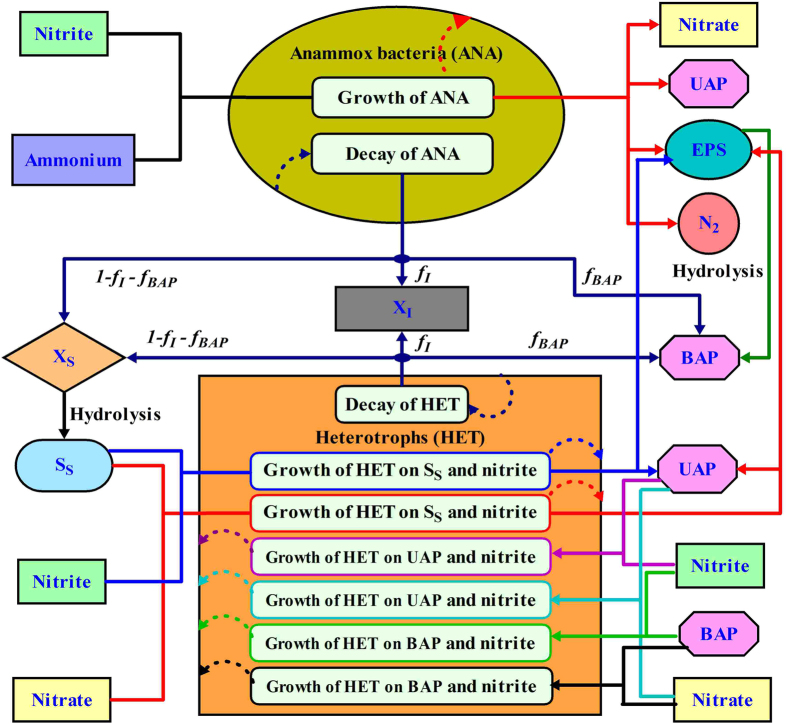
The substrate conversion and microbial interaction among ammonium (S_NH4_), nitrite (S_NO2_), nitrate (S_NO3_), readily biodegradable substrate (S_S_), anammox organisms (X_ANA_), heterotrophic biomass (X_HET_), slowly biodegradable substrate (X_S_), residual inert biomass (X_I_), utilization-associated products (S_UAP_), biomass-associated products (S_BAP_), and extracellular polymeric substances (X_EPS_).

The substrate conversion and microbial interaction among ammonia (S_NH4_), nitrite (S_NO2_), nitrate (S_NO3_), readily biodegradable substrate (S_S_), anammox organisms (X_ANA_), heterotrophic biomass (X_HET_), slowly biodegradable substrate (X_S_), residual inert biomass (X_I_), utilization-associated products (S_UAP_), biomass-associated products (S_BAP_), and extracellular polymeric substances (X_EPS_) are presented in Fig. 7. The process rate expressions of heterotrophic and anammox organisms are provided in Table S1, and the stoichiometric coefficients for these organisms are given in Table S2. The generation or utilization rate of a model component in a given biochemical process can be obtained through multiplication of related process stoichiometrics and kinetics. The values for the kinetic and stoichiometric parameters with symbols and units are given in Table S3.

### 1-D biofilm modeling approach

The structure of the 1-D biofilm model used in this work is based on the biofilm model previously reported by Wanner and Gujer^41^. Therefore, only some specific details newly introduced will be given. This 1-D model is implemented to the well-established AQUASIM simulation software^42^, which offers a free definition of the biokinetic model, flow scheme, process control strategies, graphic support of the simulation, experimental data, and communication with spreadsheet programs^40^. In this work, biofilm structures were considered as a continuum. No diffusive mass transfer of biomass within the biofilm matrix was taken into consideration. Parameters about biofilm structures and mass transfer are listed in Table S4.

Attachment of particulates and biological reaction in the bulk liquid were not considered^43^. The boundary layer thickness was set at 100 mm, irrespective of biofilm thickness. In the simulations, the model was initialized with a random distribution of biomass, with the total initial volume fraction equally split between anammox bacteria, heterotrophs, and inert biomass (Table S4). Initial conditions for all simulations assumed a biofilm thickness of 20 μm; and the concentrations of all soluble components at zero in the biofilm and bulk liquid. The biofilm was discretized into 20 grid points. The bulk volume and biofilm surface area of the reactor were 1 m^3^ and 20 m^2^, respectively. An unconfined reactor, which assumes a constant bulk volume, was adopted for keeping the specific loading rate constant and the same surface loading of nitrogen. Influent NH_4_^+^-N and NO_2_^−^-N concentrations were both set at 100 mg N L^−1^ for all simulations. Simulations were typically run for up to 200 days, which allowed for steady state conditions regarding effluent quality, biofilm thickness, and community composition. To achieve steady-state biofilm conditions (including thickness), the biofilm detachment rate was adjusted to equal the biofilm growth rate, once biofilm thickness reached the desired value^43^.

### 2-D biofilm modeling approach

The 2-D individual based model (ibM) used in this work followed the principles described in Picioreanu *et al.*^44^ and Lardon *et al.*^45^. The development of biofilm was simulated in a square computational domain defined in Cartesian coordinates. The computational domain consisted of three spatial sub-domains: the bulk liquid, the diffusion boundary layer and the biofilm. The bulk liquid sub-domain was considered completely mixed, which had a uniform distribution of the component concentrations. The boundary layer was defined at constant thickness from the biofilm surface. In the biofilm and the boundary layer sub-domains, the time-dependent concentrations of solute components were calculated from diffusion-reaction equations. Bulk concentrations of solute and particulate components were calculated from overall mass balances in the system, including inlet-outlet flows, reactions in the bulk and mass transfer to/from the biofilm. These mass balances were explained in detail in Picioreanu *et al.*^44^.

The biofilm structure was represented by a collection of discrete non-overlapping hard cylinders of solid (also called ‘particulate’) components^44^. Each biomass particle contained active biomass of a single microbial type and inert material biomass, which was surrounded by an EPS capsule produced by the biomass within the particle. Each biomass particle grew and produced EPS according to a rate defined by concentrations of solutes and particulates. The biomass particles were divided into two daughter particles once their size exceeded a critical size. An EPS-only particle was excreted if the mass fraction of EPS relative to total particle mass reached a critical value. After division and EPS excretion, particles were redistributed (‘shoved’) in order not to overlap with each other^46^. With such step sequence the whole biofilm size developed. All biomass particles growing outside the maximum biofilm size would be removed from the computational domain due to biomass detachment. In this particle-based biomass description, all microbial groups (i.e. anammox bacteria, UAP-uptaking heterotrophic bacteria (HetU), BAP-uptaking heterotrophic bacteria (HetB) and S_S_-uptaking heterotrophic bacteria (HetD)) and EPS as well were particles defined by an internal composition (mass of one or more particulate substances), by size and by location in space^46^.

## Additional Information

**How to cite this article**: Liu, Y. *et al.* Assessment of Heterotrophic Growth Supported by Soluble Microbial Products in Anammox Biofilm using Multidimensional Modeling. *Sci. Rep.* **6**, 27576; doi: 10.1038/srep27576 (2016).

## Supplementary Material

Supplementary Information
