# Supplementary material for: Assessment of Heterotrophic Growth Supported by Soluble Microbial Products in Anammox Biofilm using Multidimensional Modeling
Source: Sci Rep. 2016 Jun 7;6:27576. doi: 10.1038/srep27576 (PMC4895179; doi:10.1038/srep27576)
Supplement: Supplementary Information [file srep27576-s1.pdf]

Supplementary Materials

Assessment of Heterotrophic Growth Supported by Soluble Microbial Products in Anammox Biofilm using Multidimensional Modeling

Yiwen Liu[a,b], Jing Sun[a], Lai Peng[c], Dongbo Wang[d], Xiaohu Dai[a], Bing-Jie Ni[a,*]

[a] State Key Laboratory of Pollution Control and Resources Reuse, College of Environmental Science and Engineering, Tongji University, Shanghai 200092, PR China

[b] Centre for Technology in Water and Wastewater, School of Civil and Environmental Engineering, University of Technology Sydney, Sydney, NSW 2007, Australia

[c] Laboratory of Microbial Ecology and Technology (LabMET), Ghent University, Coupure Links 653, 9000 Ghent, Belgium

[d] College of Environmental Science and Engineering, Hunan University, Changsha 410082, China; Key Laboratory of Environmental Biology and Pollution Control (Hunan University), Ministry of Education, Changsha 410082, China

***Corresponding author:**

Dr. Bing-Jie Ni

Phone: +86 21 65986849

Fax: +86 21 65983602

E-mail: bjni@tongji.edu.cn

The following is included as additional supplementary materials for this paper:

Table S1. Process kinetic rate equations for the biological processes model

Process	Kinetics rates expressions
1. Hydrolysis	$k_H \frac{X_S / X_{HET}}{K_X + X_S / X_{HET}} X_{HET}$
2. Growth of X_{ANA}	$\mu_{ANA} \frac{S_{NH4}}{K_{NH4}^{ANA} + S_{NH4}} \frac{S_{NO2}}{K_{NO2}^{ANA} + S_{NO2}} X_{ANA}$
3. Decay of X_{ANA}	$b_{ANA} X_{ANA}$
4. Release of X_{EPS}	$k_{hyd} X_{EPS}$
5. Growth of X_{HET} on S_S and S_{NO2}	$\mu_{H,S} \eta_{NOx} \frac{S_S}{K_S + S_S} \frac{S_{NO2}}{K_{NO2}^{HET} + S_{NO2}} \frac{S_{NH4}}{K_{NH4}^{HET} + S_{NH4}} X_{HET}$
6. Growth of X_{HET} on S_S and S_{NO3}	$\mu_{H,S} \eta_{NOx} \frac{S_S}{K_S + S_S} \frac{S_{NO3}}{K_{NO3} + S_{NO3}} \frac{S_{NH4}}{K_{NH4}^{HET} + S_{NH4}} X_{HET}$
7. Growth of X_{HET} on S_{UAP} and S_{NO2}	$\mu_{H,UAP} \eta_{NOx} \frac{S_{UAP}}{K_{UAP} + S_{UAP}} \frac{S_{NO2}}{K_{NO2}^{HET} + S_{NO2}} \frac{S_{NH4}}{K_{NH4}^{HET} + S_{NH4}} X_{HET}$
8. Growth of X_{HET} on S_{UAP} and S_{NO3}	$\mu_{H,UAP} \eta_{NOx} \frac{S_{UAP}}{K_{UAP} + S_{UAP}} \frac{S_{NO3}}{K_{NO3} + S_{NO3}} \frac{S_{NH4}}{K_{NH4}^{HET} + S_{NH4}} X_{HET}$
9. Growth of X_{HET} on S_{BAP} and S_{NO2}	$\mu_{H,BAP} \eta_{NOx} \frac{S_{BAP}}{K_{BAP} + S_{BAP}} \frac{S_{NO2}}{K_{NO2}^{HB} + S_{NO2}} \frac{S_{NH4}}{K_{NH4}^{HET} + S_{NH4}} X_{HET}$
10. Growth of X_{HET} on S_{BAP} and S_{NO3}	$\mu_{H,BAP} \eta_{NOx} \frac{S_{BAP}}{K_{BAP} + S_{BAP}} \frac{S_{NO3}}{K_{NO3} + S_{NO3}} \frac{S_{NH4}}{K_{NH4}^{HET} + S_{NH4}} X_{HET}$
11. Decay of X_{HET}	$b_H X_{HET}$

Table S2. Stoichiometric matrix for the biological processes model

Variable<br>Process	S_{NH4}<br>N	S_{NO2}<br>N	S_{NO3}<br>N	S_{N2}<br>N	S_{UAP}<br>COD	S_{BAP}<br>COD	S_S<br>COD
1. Hydrolysis							1
2. Growth of X_{ANA}	$-\frac{1}{Y_{ANA}}-i_{NBM}$	$-\frac{1}{Y_{ANA}}-\frac{1}{1.14}$	$\frac{1}{1.14}$	$\frac{2}{Y_{ANA}}$	f_{UAP}		
3. Decay of X_{ANA}	$i_{NBM}-i_{NXI}f_I$					f_{BAP}	
4. Release of X_{EPS}						1	
5. Growth of X_{HET} on S_S and S_{NO2}	$-i_{NBM}$	$-\frac{(1-Y_{H,S})(1-k_{UAP}-k_{EPS})}{1.71Y_{H,S}}$		$\frac{(1-Y_{H,S})(1-k_{UAP}-k_{EPS})}{1.71Y_{H,S}}$	$\frac{k_{UAP}}{Y_{H,S}}$		$-\frac{1}{Y_{H,S}}$
6. Growth of X_{HET} on S_S and S_{NO3}	$-i_{NBM}$		$-\frac{(1-Y_{H,S})(1-k_{UAP}-k_{EPS})}{2.86Y_{H,S}}$	$\frac{(1-Y_{H,S})(1-k_{UAP}-k_{EPS})}{2.86Y_{H,S}}$	$\frac{k_{UAP}}{Y_{H,S}}$		$-\frac{1}{Y_{H,S}}$
7. Growth of X_{HET} on S_{UAP} and S_{NO2}	$-i_{NBM}$	$-\frac{1-Y_{H,UAP}}{1.71Y_{H,UAP}}$		$\frac{1-Y_{H,UAP}}{1.71Y_{H,UAP}}$	$-\frac{1}{Y_{H,UAP}}$		
8. Growth of X_{HET} on S_{UAP} and S_{NO3}	$-i_{NBM}$		$-\frac{1-Y_{H,UAP}}{2.86Y_{H,UAP}}$	$\frac{1-Y_{H,UAP}}{2.86Y_{H,UAP}}$	$-\frac{1}{Y_{H,UAP}}$		
9. Growth of X_{HET} on S_{BAP} and S_{NO2}	$-i_{NBM}$	$-\frac{1-Y_{H,BAP}}{1.71Y_{H,BAP}}$		$\frac{1-Y_{H,BAP}}{1.71Y_{H,BAP}}$		$-\frac{1}{Y_{H,BAP}}$	
10. Growth of X_{HET} on S_{BAP} and S_{NO3}	$-i_{NBM}$		$-\frac{1-Y_{H,BAP}}{2.86Y_{H,BAP}}$	$\frac{1-Y_{H,BAP}}{2.86Y_{H,BAP}}$		$-\frac{1}{Y_{H,BAP}}$	
11. Decay of X_{HET}	$i_{NBM}-i_{NXI}f_I$					f_{BAP}	

Table S2. Stoichiometric matrix for the biological processes model (continuous)

Variable<br>Process	X_S<br>COD	X_{EPS}<br>COD	X_{ANA}<br>COD	X_{HET}<br>COD	X_I<br>COD
1. Hydrolysis	-1				
2. Growth of X_{ANA}		f_{EPS}	$1-f_{UAP}-f_{EPS}$		
3. Decay of X_{ANA}	$1-f_I-f_{BAP}$		-1		f_I
4. Release of X_{EPS}		-1			
5. Growth of X_{HET} on S_S and S_{NO2}		$\frac{k_{EPS}}{Y_{H,S}}$		$1-k_{UAP}-k_{EPS}$	
6. Growth of X_{HET} on S_S and S_{NO3}		$\frac{k_{EPS}}{Y_{H,S}}$		$1-k_{UAP}-k_{EPS}$	
7. Growth of X_{HET} on S_{UAP} and S_{NO2}				1	
8. Growth of X_{HET} on S_{UAP} and S_{NO3}				1	
9. Growth of X_{HET} on S_{BAP} and S_{NO2}				1	
10. Growth of X_{HET} on S_{BAP} and S_{NO3}				1	
11. Decay of X_{HET}	$1-f_I-f_{BAP}$			-1	f_I

Table S3. Kinetic and stoichiometric parameter values used in this work

Parameter	Definition	Values	Unit	Source
Stoichiometry				
Y_{ANA}	yield coefficient for Anammox	0.159	g COD_X g^{-1} N	(1)
$Y_{H,S}$	yield coefficient for HB on S_S	0.34	g COD_X g^{-1} COD_S	(2)
$Y_{H,UAP}$	yield coefficient for HB on UAP	0.45	g COD_X g^{-1} COD_{UAP}	(2)
$Y_{H,BAP}$	yield coefficient for HB on BAP	0.45	g COD_X g^{-1} COD_{BAP}	(2)
k_{UAP}	yield coefficient for UAP of HET	0.05	g COD_{UAP} g^{-1} COD_S	(2)
k_{EPS}	yield coefficient for EPS of HET	0.18	g COD_{EPS} g^{-1} COD_S	(2)
f_{UAP}	fraction of UAP for Anammox growth	0.14	g COD_{UAP} g^{-1} N	(3)
f_{EPS}	fraction of EPS for Anammox growth	0.09	g COD_{EPS} g^{-1} N	(3)
i_{NBM}	Nitrogen content of biomass	0.07	g N g^{-1} COD_X	(4)
i_{NXI}	Nitrogen content of X_I	0.02	g N g^{-1} COD_X	(4)
f_I	fraction of X_I in biomass decay	0.08	g COD_X g^{-1} COD_X	(5)
f_{BAP}	fraction of BAP in biomass decay	0.0215	g COD_{BAP} g^{-1} COD_X	(6)
η_{NOx}	anoxic reduction factor	0.60	—	(4)
Kinetics				
μ_{ANA}	maximum growth rate of Anammox	0.0029	h^{-1}	(3)
b_{ANA}	death rate coefficient of Anammox	0.000125	h^{-1}	(1)
k_{hyd}	EPS hydrolysis rate coefficient	0.0071	h^{-1}	(2)
$\mu_{H,S}$	maximum growth rate of HET on S_S	0.25	h^{-1}	(5)
$\mu_{H,UAP}$	maximum growth rate of HET on UAP	0.13	h^{-1}	(3)
$\mu_{H,BAP}$	maximum growth rate of HET on BAP	0.0029	h^{-1}	(2)
b_H	death rate coefficient of HET	0.026	h^{-1}	(5)
k_H	hydrolysis rate constant	0.125	h^{-1}	(4)
K_X	hydrolysis saturation constant	1.0	g COD_X g^{-1} COD_X	(4)
K_{NH4}^{ANA}	S_{NH4} affinity constant for Anammox	0.07	g N m^{-3}	(1)
K_{NO2}^{ANA}	S_{NO2} affinity constant for Anammox	0.05	g N m^{-3}	(1)
K_{NH4}^{HET}	S_{NH4} affinity constant for HET	0.01	g N m^{-3}	(4)
K_{NO2}^{HET}	S_{NO2} affinity constant for HET	0.5	g N m^{-3}	(4)
K_{NO3}	S_{NO3} affinity constant for HET	0.5	g N m^{-3}	(4)
K_S	biomass affinity constant for S_S	2.0	g COD_S m^{-3}	(4)
K_{UAP}	biomass affinity constant for UAP	100	g COD_{UAP} m^{-3}	(2)
K_{BAP}	biomass affinity constant for BAP	85	g COD_{BAP} m^{-3}	(2)

Sources: (1) Strous et al., 1998; (2) Laspidou and Rittmann, 2002; (3) Ni et al., 2012; (4) Gujer et al., 1999; (5) Henze et al., 1987; (6) Jiang et al., 2008

Table S4. Biofilm and mass transfer parameters

Parameter	Description	Value	Reference
Biofilm			
L_f	Biofilm thickness, um	1000	This study
θ	Biofilm porosity, m^3 liquid m^{-3} biofilm	0.75	(7)
Mass transfer			
D^{Ss}	Effective diffusivity of Ss, dm^2 h^{-1}	0.000576	(8)
D^{NH4}	Effective diffusivity of NH_4^+, dm^2 h^{-1}	0.000625	(9)
D^{NO2}	Effective diffusivity of NO_2^-, dm^2 h^{-1}	0.000583	(9)
D^{NO3}	Effective diffusivity of NO_3^-, dm^2 h^{-1}	0.000583	(9)
D^{N2}	Effective diffusivity of N_2, dm^2 h^{-1}	0.000917	(9)
D^{UAP}	Effective diffusivity of UAP, dm^2 h^{-1}	0.000575	(10)
D^{BAP}	Effective diffusivity of BAP, dm^2 h^{-1}	0.000575	(10)
Density of solid phase			
ρ_{XHET}	Density of X_H, g COD m^{-3}	50000	(11)
ρ_{XI}	Density of X_I, g COD m^{-3}	50000	(11)
ρ_{XANA}	Density of X_{AN}, g COD m^{-3}	50000	(11)
ρ_{XEPS}	Density of X_{AN}, g COD m^{-3}	50000	(11)

Sources: (7) Koch et al., 2000; (8) Beun et al., 2001; (9) Picioreanu et al., 1997; (10) Laspidou and Rittmann, 2004; (11) Rittmann and McCarty, 2001

References

Beun, J.J, Heijnen, J.J, van Loosdrecht, M.C.M. 2001. N-removal in a granular sludge sequencing batch airlift reactor. Biotechnol. Bioeng. 75, 82-92.

Gujer, W., Henze, M., Mino, T., Van Loosdrecht, M. 1999. Activated sludge model no. 3. Water Science and Technology, 39(1), 183-193.

Henze, M., Grady Jr, C. P. L., Gujer, W., Marais, G. V. R., Matsuo, T. 1987. Activated Sludge Model No. 1: IAWPRC Scientific and Technical Report No. 1. IAWPRC, London.

Jiang, T., Myngheer, S., De Pauw, D. J., Spanjers, H., Nopens, I., Kennedy, M. D.,

Vanrolleghem, P. A. 2008. Modelling the production and degradation of soluble microbial products (SMP) in membrane bioreactors (MBR). Water Research, 42(20), 4955-4964.

Koch, G., Egli, K., van der Meer, J.R., Siegriest, H. 2000. Mathematical modeling of autotrophic denitrification in a nitrifying biofilm of a rotating biological contactor. Water Sci. Technol. 41, 191-198.

Laspidou, C. S., Rittmann, B. E. 2002. Non-steady state modeling of extracellular polymeric substances, soluble microbial products, and active and inert biomass. Water Research, 36(8), 1983-1992.

Laspidou, C.S., Rittmann, B.E. 2004. Modeling the development of biofilm density including active bacteria, inert biomass, and extracellular polymeric sub- stances. Water Res. 38, 3349-3361.

Ni, B.J., Ruscalleda, M., Smets, B.F. 2012. Evaluation on the microbial interactions of anaerobic ammonium oxidizers and heterotrophs in Anammox biofilm. Water Res. 46, 4645-4652.

Picioreanu, C., van Loosdrecht, M.C.M., Heijnen, J.J. 1997. Modelling the effect of oxygen concentration on nitrite accumulation in a biofilm airlift suspension reactor. Water Sci. Technol. 36, 147-156.

Rittmann, B.E., McCarty, P.L. 2001. Environmental Biotechnology, Principles and Applications. McGraw-Hill, New York.

Strous, M., Heijnen, J. J., Kuenen, J. G., Jetten, M. S. M. 1998. The sequencing batch reactor as a powerful tool for the study of slowly growing anaerobic ammonium-oxidizing microorganisms. Applied microbiology and biotechnology, 50(5), 589-596.
